# Plant-Based (Hemp, Pea and Rice) Protein–Maltodextrin Combinations as Wall Material for Spray-Drying Microencapsulation of Hempseed (*Cannabis sativa*) Oil

**DOI:** 10.3390/foods9111707

**Published:** 2020-11-20

**Authors:** Marcin Andrzej Kurek, Anubhav Pratap-Singh

**Affiliations:** 1 Food Nutrition and Health Program, Faculty of Land and Food Systems, The University of British Columbia, Vancouver, BC V6T 1Z4, Canada; marcin_kurek@sggw.edu.pl; 2 Department of Technique and Food Development, Institute of Human Nutrition Sciences, Warsaw University of Life Sciences, 02-787 Warsaw, Poland; 3 Department of Biotechnology and Food Science, Norwegian University of Science and Technology (NTNU), 7012 Trondheim, Norway

**Keywords:** microencapsulation, spray drying, maltodextrin, plant-based protein, hempseed oil

## Abstract

Conscious consumers have created a need for constant development of technologies and food ingredients. This study aimed to examine the properties of emulsions and spray-dried microcapsules prepared from hempseed oil by employing a combination of maltodextrin with hemp, pea, and rice protein as carrier materials. Oil content in the microcapsules was varied at two levels: 10 and 20%. Increasing oil load caused a decrease in viscosity of all samples. Consistency index of prepared emulsions was calculated according to Power Law model, with the lowest (9.2 ± 1.3 mPa·s) and highest values (68.3 ± 1.1 mPa·s) for hemp and rice protein, respectively, both at 10% oil loading. The emulsion stability ranged from 68.2 ± 0.7% to 88.1 ± 0.9%. Color characteristics of the microcapsules were defined by high L* values (from 74.65 ± 0.03 to 83.06 ± 0.03) and low a* values (−1.02 ± 0.015 to 0.12 ± 0.005), suggesting that the materials were able to coat the greenish color of the hemp seed oil acceptably. The highest encapsulation efficiency was observed in samples with rice protein, while the lowest was with hemp protein. Combination of maltodextrin and proteins had a preventive effect on the oxidative stability of hempseed oil. Oil release profile fitted well with the Higuchi model, with hempseed oil microencapsulated with pea protein–maltodextrin combination at 10% oil loading depicting lowest oil release rates and best oxidative stability.

## 1. Introduction

Nowadays, there is an increasing demand for healthy food products with high nutrient value, which has compelled the food industry to continually seek innovative methods of processing and preservation [1]. There is still a great concern about consuming mono- and polyunsaturated fatty acids. Hempseed (*Cannabis sativa*) oil is considered to be one of the most nutritionally healthy oils, with a well-balanced ratio (3:1) of two PUFAs (polyunsaturated fatty acids) considered essential for human nutrition: linoleic and linolenic acids [2]. However, the application of hempseed oil in food technology is still limited because of its’ low smoke point, intensive color, and flavor; thus, there is a need to explore new ways of incorporating hempseed oil into the human diet [3]. The availability of this material is also growing due to the increased acceptability of the hemp industry in the 21st century. Therefore, application of hempseed oil is more economically reasoned for ensuring sustainability of the emerging hemp and cannabis sector.

One major problem with highly nutritive oils is the formation of oxidative products, which cause generation of undesirable flavors and rancidity [4]. Hempseed oil is more prone to oxidation due to its unsaturated fatty acid content, which could be inhibited by using stabilizers or plant extracts [5]. However, such additives may or may not be desirable for food industry due to labeling requirements and consumer concerns regarding chemical additives. Microencapsulation has been proposed as a method for preventing oil oxidation, while increasing its’ nutritional value and shelf life [6]. Microencapsulation is a process of coating an active core material with a suitable wall material and can be applied to both solid particles and microscopic droplets. Several crucial factors influence the final quality of microcapsules, but most important ones are drying conditions and feed emulsion composition [7]. The most economical and industrially common technique of drying is spray-drying, which has also been used for micro-encapsulation [8]. The wall material for encapsulation should have good emulsification properties and perform as an excellent protective agent during the storage of microcapsules. Often, these requirements cannot be met with a single wall material. Thus, combination of polysaccharides and proteins, or a combination of a few wall materials with similar origins but different properties, are often used. One of the most common polysaccharides used during spray-drying microencapsulation is maltodextrin because of its low viscosity at high concentration, good solubility, adequate protection against oxidation, relatively low cost, and neutral aroma and taste [9].

However, maltodextrin has a low emulsifying capacity and emulsion stability. Therefore, a combination of maltodextrin and other compounds could be employed for the purpose of microencapsulation. Proteins are generally perceived as suitable wall materials and emulsifiers due to their amphiphilic nature [10]. Due to the growing interest of non-GMO (Genetically Modified Organisms) products and vegan diets, there is a need to search for new sources of plant-based proteins that are not negatively associated with consumer perception. While hempseed protein has shown potential to be a good source of nutritious protein, there is limited information on its potential use as an ingredient to formulate high-quality foods [11]. There is an interest in research into the health benefits of bioactive peptides prepared from hemp protein, as well as its technological functionality, such as foaming, emulsifying, gelling, and film-forming capabilities. Another potential plant-based protein source is pea protein, but it is limited in terms of methionine and tryptophan [12]. However, this protein is also inexpensive, hypoallergenic, and has no issues around consumer perception. Rice protein is also an essential source of protein, as it is a by-product derived from rice bran. The nutritional profile of rice protein is generally perceived as positive, but the emulsifying properties still need better understanding [13], especially in terms of its’ role in emulsifying hempseed oil emulsions.

Very little information is available in the contemporary literature about the microencapsulation of hempseed oil with a combination of maltodextrin and plant proteins. Therefore, this study aimed to examine the emulsion properties and the properties of spray-dried microencapsulated powders prepared with hempseed oil using a combination of maltodextrin with hemp, pea, and rice proteins as coating materials.

## 2. Materials and Methods

### 2.1. Materials

Commercial protein powders of hemp, pea, and rice used as wall materials were supplied by Yupik (Montreal, QC, Canada). The protein content was 50% for the hemp protein and 80% for each of the pea and rice protein powders. In the rice protein, other compounds incorporated were 5% lipids and 5% carbohydrates such as dietary fiber. Pea protein had 8% lipids and 2% carbohydrates, with 1% dietary fiber. The hemp protein powder was processed with an isoelectric precipitation technique to enlarge the protein content up to 80% using a method suggested by Hadnađev et al. (2018) [14]. Other compounds were lipids, 7.5%, and carbohydrates, 2.1%. Maltodextrin was provided by a local supplier (solubility 586 g/L, DE 10-12). Hempseed oil was supplied by Manitoba Harvest Hemp Foods (Winnipeg, MB, Canada). All reagents used in the study were HPLC grade supplied by Thermo Fisher Scientific, Waltham, Canada.

### 2.2. Emulsion Preparation and Spray-Drying Micro-Encapsulation

An oil-in-water emulsion with a different oil load (10 and 20% w/w solids) was prepared by first dissolving the respective proteins in a sodium phosphate buffer with a pH of 6.5 that was then solubilized by constant stirring for 2 h. Then, the mixture combined with solubilized maltodextrin and mixed. The ratio of protein to maltodextrin was kept constant at 1:1 through all the experiments, as the optimal ratio was based on our preliminary studies. The content of solid compounds in all the emulsions was kept at 20%, on the basis of previous research [15]. Hempseed oil was then added to the wall material suspension and homogenized at 10,000 rpm using a rotor-stator homogenizer (Polytron PCU-2-110 homogenizer, Brinkmann Instruments, Inc., Westbury, NY, USA) for 5 min at 25 °C.

The emulsions were dried using a spray-drier Buchi B-290 (Switzerland) equipped with a two-fluid nozzle of a diameter of 0.7 mm. Prepared emulsions were fed into the drying chamber using a peristaltic pump, operating at a speed of 7.5 mL/min, and a drying airflow of 600 L/h with pressure drop 0.75 bar. The inlet temperature was 160 °C, and the outlet temperature was kept at 90 °C. Dried powders were collected, closed hermetically, and stored at −20 °C before further examination.

### 2.3. Emulsion Properties

The viscosity of the emulsion was evaluated using a rotational rheometer (Modular Compact Rheometer, MCR 302, Anton Paar, Graz, Austria) using a plate–plate system with a 1.0 mm gap between the plates. Measurements were performed in triplicate at a constant temperature (25 °C). The samples were subjected to an increasing shear rate, from 0 to 1000^−1^. The viscosity was recorded and assessed using the Power Law model, as shown in Equation (1):

(1) $$\tau =K\gamma^{n}$$

where τ  is the shear stress (Pa), *K* is the consistency index, γ is the shear rate, and *n* is the flow behavior index.

The droplet size of emulsions was determined using a Litesizer 500 (Anton Paar, Austria). A total of 1 mL of the emulsion was diluted with 200 mL of water, and then placed in the cuvette for a light scattering determination of particle size. The results were expressed as a De Brouckere (D 4,3) mean diameter. The span was assessed using Equation (2):

Span = D_50_/(D_90_ − D_10_), (2)

D_x_ refers to the particle size, for which x% of the particles are smaller, and with particle sizes expressed in micrometers. Measurements were carried out in triplicate at a temperature of 25 °C.

Stability of the emulsion was determined using the separation storage method discussed earlier [16]. Briefly, immediately after preparation, 15 mL of the emulsion was transferred to a graduated cylinder, and the volume of the upper phase was measured after 24 h. The stability was measured as the percentage separation of the upper phase height to the initial height.

### 2.4. Microcapsules Characteristics

#### 2.4.1. Moisture Content

Moisture content was determined by the gravimetric method by drying in an oven at 105 °C until constant weight and was expressed as a percentage. Measurements were conducted in triplicate.

#### 2.4.2. Color Measurement

Color of the microcapsules was assessed using a LabScan XE (HunterLab, Reston, VA, USA) and presented as L*, a*, b* according to CIELab. The following parameters were determined and tested: L * (L = 0 (black), L = 100 (white)), a * (−a = green, +a = red), and b * (−b = blue, +b = yellow). Measurements were conducted in triplicate.

#### 2.4.3. Particle Size of Microcapsules

Microcapsules were examined in terms of the particle size using a particle size analyzer (Litesizer 500, Anton Paar, Austria). Emulsions were dissolved in a 0.5% SDS solution in an amount equal to 1% w/v. For measurements of microcapsules, we used ethyl alcohol 96% as a solvent. Then, 0.75 mL of suspension was examined using a light scattering determination of the particle size. Results were expressed as a De Broucker (D 4,3) mean diameter. Polydispersity index was also calculated using a similar equation as Equation (2).

#### 2.4.4. Encapsulation Efficiency

Encapsulation efficiency was determined using a method based on the difference between the total oil and surface oil. To examine the surface oil content (*S_o_*), we mixed 0.75 g of microcapsules with 10 mL of hexane for 2 min.

Then, the suspension was filtered through a Whatman filter paper (no°4), and the powder collected on the filter was rinsed 3 times with 10 mL of hexane. The solvent was evaporated using a vacuum rotary evaporator at 45 °C until constant weight. *S_o_* was determined by the mass difference between the initial clean flask and the one containing extracted oil residue. Total oil (*T_o_*) was assumed to be equal to the initial oil.

The encapsulation efficiency (*EE*) was calculated using Equation (3):

(3) $$EE\;\left(\%\right)=\frac{T_{o}-S_{o}}{T_{o}}.$$

#### 2.4.5. Oxidative Stability

Oxidative stability of hempseed oil microcapsules was examined using 2 methods: peroxide value (PV) and thiobarbituric acid assay (TBA), after storage of 5 g of powder at 60 °C for 30 days in absence of light in closed 50 mL Falcon tubes.

Peroxide value was determined by employing the following extraction. A total of 0.5 g of sample powder was mixed for 30 min with 5 mL of water. After that, 600 µL was vortexed with a mixture of 3 mL of hexane and isopropanol (ratio 2:1) at 1000 rpm for 10s. Suspension was centrifuged at 1000× *g* for 4 min. Then, 200 µL was taken from the supernatant and treated according to the method of Shantha and Decker [17]. Values were presented as milliequivalents of peroxide per kilogram of oil.

TBA assay was conducted by employing the method of Giorgio et al. (2019) [18]. A powdered sample (0.5 g) was shaken with 1.78 mL of 5% w/v trichloroacetic acid (TCA) for 30 min. After that, the sample was centrifuged (10,000× *g*, 10 min) to obtain a supernatant without particles. A total of 0.5 mL of supernatant was mixed with 0.5 mL of 0.5% w/v TBA solution and closed in tubes. The mixture was incubated with shaking at 70 °C. Absorbance was measured at 523 nm using a Shimadzu spectrophotometer (Shimadzu UV-1800, Shimadzu, Kyoto, Japan). TBA values were presented as milligrams of malondialdehyde/kg of oil according to Equation (4):

(4) $$\mathrm{TBA}=\;\frac{\mathrm{Abs}*M*\mathrm{Vs}*\mathrm{Ve}*\;1000}{\varepsilon *l*m}$$

where Abs is the absorbance at 532 nm, M is the malondialdehyde molar mass (72 g/mol), Vs is the sample volume (0.5 mL), Ve is the extract volume (1.78 mL), ε is the molar extinction coefficient of the colored complex (1.56 × 10^5^ M^−1^), l is the optical path (1 cm), and m is the sample mass (g).

#### 2.4.6. Release of Oil

The release of oil from microcapsules was evaluated using an aqueous model (phosphate buffer solution (PBS) in 6.5). Microcapsules (2.5 g) were closed in bags formed from paper filters. Then, they were closed with 100 mL of PBS and shaken at 55 rpm at 30 °C. Aliquots (15 mL) were removed (in triplicate) at specific time intervals, and the hemp oil released was extracted 3 times with hexane by liquid–liquid extraction. Addition of the corresponding buffer maintained the initial volume. Next, hexane was removed using a rotatory evaporator (R-100 Büchi, Flawil, Switzerland) at 40 °C until constant weight. The obtained data were fitted to the Higuchi model, as presented in Equation (5) provided by Pratap-Singh et al. [8].

(5) $$\frac{M_{t}}{M_{\infty }}=k\;\times t^{1/2}$$

*M_t_* is defined as the quantity of oil released at any time, *t*. The release rate constants (*k*) were obtained from the slope of a plot *M_t_*/*M*_∞_ versus (time)^1/2^.

#### 2.4.7. Scanning Electron Microscopy

Particle size and morphology were evaluated by scanning electron microscopy (SEM). Powders were mounted on aluminum stubs using double-sided tape and were coated with a thin gold layer. SEM images were acquired with a scanning electron microscope (Hitachi S2600 variable pressure VP-SEM, Hitachi High-Technologies, Tokyo, Japan) under a low vacuum with a 20 kV acceleration voltage.

### 2.5. Statistical Analysis

Results were expressed as mean ± standard deviation and were analyzed by ANOVA. Means were tested with Tukey’s post hoc analysis using Statistica 13.0 software (StatSoft, St Tulsa, OK USA) for testing significant differences with α = 0.05.

## 3. Results and Discussion

### 3.1. Properties of Hempseed Oil Emulsions

Emulsions of hempseed oil using a mixture of maltodextrin with various proteins were examined using standardized methods for assessing their physical properties. In all samples, it was observed that increasing the oil load from 10 to 20% decreased the viscosity of the emulsion (Table 1). This tendency is similar to the observation made by Di Giorgio et al. [18]. However, taking into consideration the flow behavior of emulsions with increasing shear stress, we could observe that the consistency coefficient had the lower value in the samples with hemp protein at 10 and pea protein with 20% oil load, followed by pea protein with 10% oil load and hemp protein at 20% oil load; with both 10% and 20% oil loading of rice protein yielding the highest values of consistency coefficient. Flow behavior revealed that the samples had non-Newtonian and pseudoplastic properties, mainly observed in the samples with rice protein and 10% oil load. The samples with hemp and pea protein, and an oil load of 10%, had a flow behavior value of nearly 1, meaning that they were nearly Newtonian fluids. At 20% oil load, rice protein emulsions became more Newtonian, while pea and hemp protein emulsions became more non-Newtonian. Following the same trend as flow behavior, droplet sizes of rice protein increased on increasing the oil loading from 10% to 20%, while that of pea and hemp protein decreased. Moreover, we observed that there was a correlation between the oil load and span. When the oil content increased, span decreased in all the samples, which means that the distribution of particle size was more uniform in the samples with more oil load [19]. We observed that higher oil content resulted in a decrease in emulsion stability. This is because at a higher oil content, there is insufficient protein to properly stabilize oil droplets, and as such, a weaker emulsion with a lower ability to defend against coalescence during storage was formed [20].

There is a general theory in research that the smaller the droplets, the higher the emulsion stability. In our study, this theory was not supported by the results of the pea and hemp proteins as wall material; on the other hand, they were found to have a smaller particle size and lower emulsion stability at higher oil concentrations. This may be attributed to the fact that emulsion stability and emulsion viscosity are often interdependent. As stated in works by other authors, it is proven that shear-thinning properties could indicate emulsion stability, and a higher viscosity limits the movement of oil droplets, which prevents them from coalescence [15]. The second explanation of the variation of emulsion stability could be the differences in zeta potential, which could vary in an emulsion on the basis of the protein and could lead to poor physical stability [21].

### 3.2. Physical Properties of Microcapsules with Hempseed Oil

Moisture content of the samples varied between 2.16% and 4.20% (Table 2). Highest differences in moisture content was observed for rice protein microcapsules (2.16 ± 0.06% and 4.20 ± 0.08% for 10 and 20% oil load, respectively) (Table 2). The higher the oil content, the higher the moisture content for all samples, which may be attributed to the fact that at a higher oil content, more water was held in the emulsion due to the hydration capacity and binding effects of protein with oil and water. Therefore, this water might not have been easily removed by the spray-drying process. Nevertheless, obtained moisture content was lower than 4%, which could be perceived as a dry powder in terms of definition for the food industry [22].

Samples were mild beige in color, with little differences between each other. In hempseed protein–maltodextrin samples, there was no statistical difference (*p* > 0.05) between 10 and 20% oil load. Pea protein–maltodextrin samples had the lowest lightness at 10% oil content. No definite trend was observed in terms of a* parameter, as it ranged from −1.02 up to 0.12. However, these results are still small in nominal values to be observed by the human eye. It is important to note that the wall in all samples was thick enough to coat hempseed oil, a greenish colored oil. Parameter b* ranged between 10.96 and 17.99, with rice and pea protein–maltodextrin microcapsules being more yellow at higher oil content. Particle size is also an important parameter that affects the flowability, compressibility, bulk density, and oxidative stability of the microcapsules [4]. The particle size of microcapsules was higher than the droplet size of their emulsions, which is a typical observation due to the addition of wall material. The smallest particle size was observed in samples with rice protein as the wall material and highest in the pea sample with 20% oil load. Pea protein–maltodextrin microcapsules had most uniform particle size, as their span was 0.56 in the samples with 10% oil content.

### 3.3. Morphology of Microcapsules

**Figure 1:**
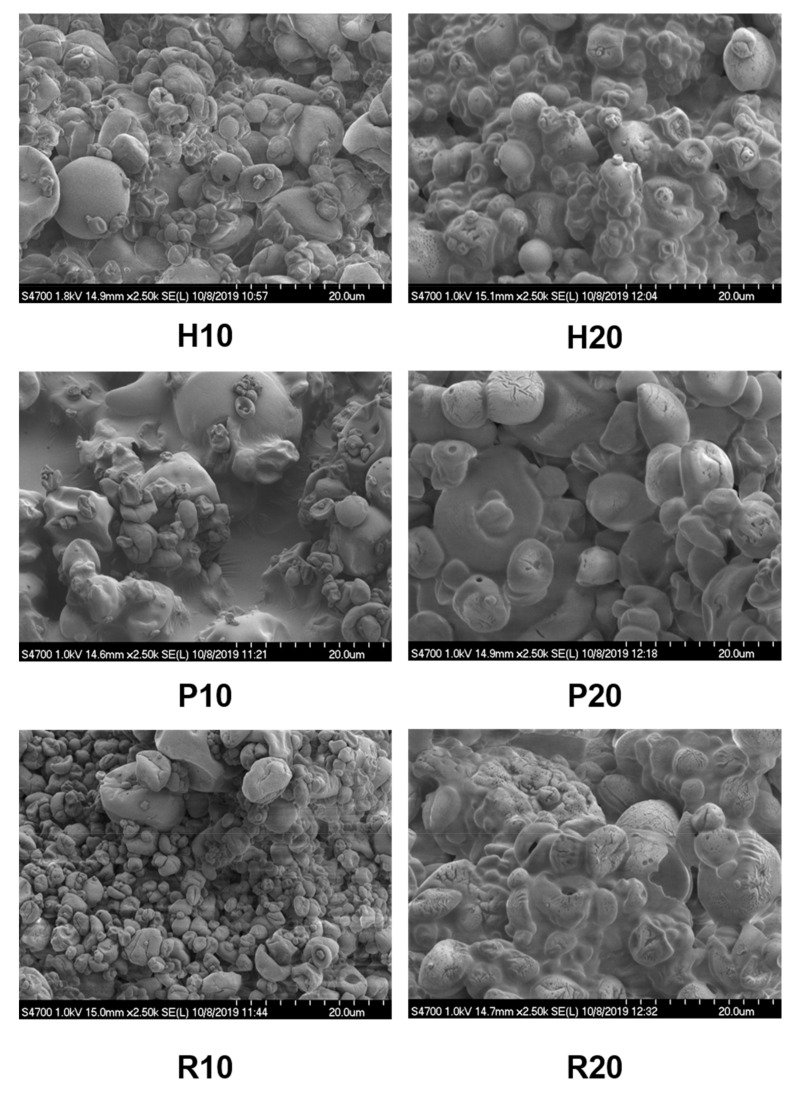
Protein–maltodextrin-coated hempseed oil microcapsules observed with SEM.

Figure 1 displays SEM images of the microcapsules, which were prepared with different oil load. All microcapsules were either spherical or nearly spherical, which is a standard trait for samples prepared by spray-drying [23]. Generally, hempseed and rice samples, with a 10% oil load, had a smoother surface than samples with 20%. This could be caused by the increased share of protein in the composition, wherein the oil content was smaller. However, Loksuwan reported that the smooth surface of microencapsulated β-carotene with maltodextrin is related to the low molecular weight of the sugar content of maltodextrin, but there is no consensus in this aspect [24]. Nevertheless, a smoother surface may indicate better retention of the core inside the capsules [15]. The application of proteins as wall material generally could lead to more extensive variations and non-uniformity in the morphology of particles [25].

### 3.4. Encapsulation Efficiency and the Level of Oxidation

Encapsulation efficiency is one of the most vital criteria in terms of assessing the microencapsulation process, and corresponding microencapsulation efficiency of our samples are reported in Table 3. The lowest encapsulation efficiency was observed for hempseed protein–maltodextrin microcapsules at 20% oil content (37.12 ± 0.31%), whereas the highest was for the rice protein–maltodextrin combination at 10% oil content (79.37 ± 1.11%). The novelty of applying hempseed oil as a core material in encapsulation with spray drying made it challenging to compare the results. However, Aberkane et al. [26] investigated pea protein–pectin combination for encapsulating polyunsaturated fatty acid-rich oil by spray drying, and concluded that pea protein was not a satisfactory wall material for maximizing encapsulation efficiency and minimizing lipid oxidation without addition of any polysaccharide. Encapsulation efficiency of the combination of maltodextrin and proteins is influenced by the ratio of both these wall materials. Generally, maltodextrin lacks surface-active properties, and this makes them inferior wall materials. Therefore, adding protein is an efficient way of increasing encapsulation efficiency. This is typically caused by the unfolding and adsorption of oil–water interfaces, changing protein structures, and causing a resistant and stable layer over the oil droplets [27]. Moreover, encapsulation efficiency could be explained by the amino acid profile and the conformation of the protein. The rice protein has four significant fractions: albumin (5–10%), globulin (7–17%), glutein (75–81%), and prolamine (3–6%) [28]. Gluteins are not soluble in water, which could lead to higher encapsulation efficiency, as a similar tendency has been observed by other researchers [23]. This could explain the high difference of encapsulation efficiency between 10% and 20% oil load in samples with rice protein (nearly 18%).

Samples with hempseed oil as a core were assessed using PV and TBA after storage at 60 °C for 30 days. In both measurements, values of oxidation were lower when the oil content was lower. Highest PV was observed for the pea protein–maltodextrin microcapsules with 20% oil content (4.65 ± 0.019 meq/kg oil), while the lowest was observed in the same sample with 10% oil content (2.76 ± 0.011 meq/kg oil). Both values were lower than the PV of oil stored without being encapsulated (16.04 meq/kg oil). PV is the measurement of the number of oxidative products such as peroxides and hydroperoxides. They are very reactive and could decompose or polymerize, producing intermediate and secondary oxidative products. Oxidative reactions of PUFAs could lead to unacceptable sensory properties [29]. TBA value for hempseed oil store without encapsulation was 2.02 mg MDA (malondialdehyde)/kg oil. The highest content of TBA was observed for the rice protein–maltodextrin microcapsules at 20% oil load, while lowest TBA was observed for pea protein samples at 10% oil load. All samples showed a higher TBA at higher oil load and vice-versa. TBA values were similar to the results obtained by Avramenko et al. [30]. Thus, it is seen that encapsulation of hempseed oil could lead to inhibition of oxidative reactions in terms of PV and TBA. Still, there is a need to optimize the encapsulation technique to lower this oxidation rate, which might include incorporation of plant-based saponins as natural surfactants to augment the effect of plant-based proteins and maltodextrin combinations [31].

**Figure 2:**
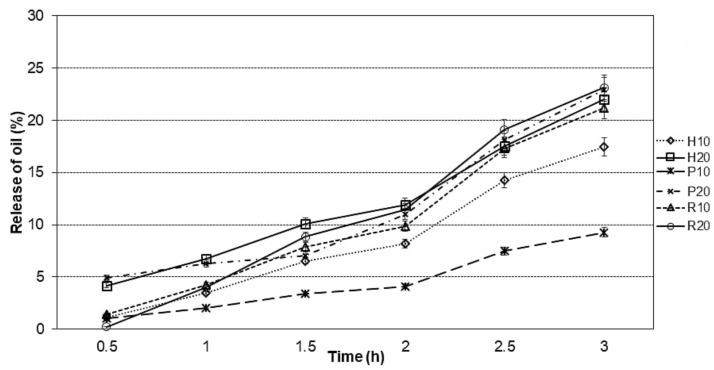
Oil release from hempseed oil microcapsules encapsulated with combination of proteins with maltodextrin.

Oil release was compared between the samples following a 3-h release study. This approach aimed to explore how microcapsules would behave in a model food condition. As shown in Figure 2, after 3 h, lowest release was observed in pea protein–maltodextrin microcapsules at 10% oil content (9.27%), and the highest in samples with 20% oil content and rice protein (23.15%). The release profile fitted well with the Higuchi model based on the correlation coefficient (*R^2^*), which was higher than 97% [19]. It occurred that a smaller oil content caused lower release of oil from the samples (Table 3), with pea protein samples having the lowest release rate constant at 10% oil load and rice protein samples having highest release rate constant at 20% oil load.

## 4. Conclusions

Two levels of oil load were examined in terms of emulsification and microencapsulation. Rheological measurements revealed differences in the viscosity of samples and how they behaved in increasing the shear rate. Increasing oil load in the sample led to a decrease in viscosity. The moisture content was at a satisfactory level for dry products in all of the samples. Hempseed protein was not efficient in terms of encapsulation efficiency. Microencapsulation of hempseed oil with plant-based protein–maltodextrin complex successfully inhibited oxidation by limiting the PV and TBA of the oil within 5 meq/kg oil and 1 mg MDA/kg oil, respectively, for all samples. Both rice and pea proteins were found to be a viable plant-based alternative as wall material, with rice protein microcapsules having less than 20 μm particle size and ≈80% encapsulation efficiency at 10% oil loading, and pea protein microcapsules depicting the lowest release rate constants and highest oxidative inhibition at 10% oil loading. These results can be used as a reference for further studies in hempseed oil microencapsulation to achieve desired physical and chemical properties.

## Figures and Tables

**Table 1:** Rheological properties, droplet size, and stability of emulsions prepared with hempseed oil and combination of proteins with maltodextrin.

Origin of Protein	Initial Oil Content (%)	Viscosity (at 206 s^−1^ [mPa·s])	K [mPa·s] (Consistency Index)	n (Flow Behavior Index)	Droplet Size (µm)	Span	Emulsion Stability (%)
Hemp	10	5.6 ± 0.8 ^b^	9.2 ± 1.3 ^a^	0.91 ± 0.061 ^c^	12.71 ± 1.698 ^b^	0.93 ± 0.001 ^d^	76.95 ± 1.65 ^c^
	20	5.15 ± 0.25 ^b^	17.4 ± 0.8 ^b^	0.85 ± 0.122 ^d^	10.97 ± 1.297 ^a^	0.59 ± 0.115 ^b^	68.22 ± 0.71 ^a^
Rice	10	6.85 ± 0.25 ^c^	68.3 ± 1.1 ^d^	0.55 ± 0.006 ^a^	10.28 ± 0.001 ^a^	0.86 ± 0.002 ^c^	86.61 ± 1.01 ^d^
	20	4.55 ± 0.15 ^a^	28.15 ± 1.05 ^c^	0.76 ± 0.108 ^b^	18.79 ± 0.005 ^c^	0.52 ± 0.005 ^a^	72.30 ± 1.11 ^b^
Pea	10	8.6 ± 0.5 ^d^	11.55 ± 1.45 ^a^	0.94 ± 0.04 ^d^	18.14 ± 0.002 ^c^	0.84 ± 0.003 ^c^	88.15 ± 0.95 ^d^
	20	6.65 ± 0.65 ^c^	9.85 ± 0.55 ^a^	0.86 ± 0.024 ^c^	12.04 ± 3.087 ^b^	0.62 ± 0.058 ^b^	75.75 ± 0.55 ^c^

^a–d^—different letters mean significant differences between the samples within the column (*p* ≤ 0.05).

**Table 2:** Physical properties of hempseed oil microcapsules encapsulated with combination of proteins with maltodextrin.

Origin of Protein	Initial Oil Content (%)	Moisture Content (%)	L*	A*	B*	Particle Size (µm)	Span (Dry)
Hemp	10	2.59 ± 0.025 ^b^	74.65 ± 0.03 ^a^	−0.35 ± 0.005 ^d^	15.09 ± 0.015 ^c^	21.10 ± 0.04 ^c^	1.18 ± 0.362 ^c^
	20	2.83 ± 0.005 ^c^	74.75 ± 0.025 ^a^	−1.02 ± 0.015 ^a^	16.65 ± 0.002 ^d^	33.90 ± 1.25 ^d^	0.75 ± 0.101 ^b^
Rice	10	2.16 ± 0.060 ^a^	80.64 ± 0.03 ^d^	0.12 ± 0.005 ^f^	10.96 ± 0.005 ^a^	11.71 ± 0.41 ^a^	1.59 ± 0.296 ^d^
	20	4.20 ± 0.080 ^e^	77.95 ± 0.06 ^b^	−0.55 ± 0.002 ^c^	17.03 ± 0.001 ^e^	19.92 ± 1.99 ^b^	0.76 ± 0.036 ^b^
Pea	10	2.68 ± 0.210 ^b^	83.06 ± 0.025 ^e^	−0.12 ± 0.011 ^e^	12.28 ± 0.005 ^b^	34.54 ± 4.44 ^d^	0.56 ± 0.099 ^a^
	20	3.09 ± 0.075 ^d^	78.63 ± 0.005 ^c^	−0.76 ± 0.005 ^b^	17.99 ± 0.005 ^e^	56.23 ± 4.02 ^e^	0.89 ± 0.296 ^c^

^a–d^—different letters mean significant differences between the samples within the column (*p* ≤ 0.05).

**Table 3:** Encapsulation efficiency, oxidation, and oil release from hempseed oil microcapsules encapsulated with combination of proteins with maltodextrin.

Origin of Protein	Initial Oil Content (%)	Encapsulation Efficiency (%)	PV (meq/kg Oil)	TBA (mg MDA/kg Oil)	Release of Oil (k)
Hemp	10	48.13 ± 0.902 ^b^	2.94 ± 0.012 ^b^	0.82 ± 0.011 ^b^	6.63 ± 0.12 ^b^
	20	37.12 ± 0.311 ^a^	4.37 ± 0.016 ^b^	0.98 ± 0.017 ^c^	7.04 ± 0.04 ^c^
Rice	10	79.37 ± 1.113 ^d^	2.89 ± 0.021 ^a^	0.97 ± 0.011 ^c^	7.41 ± 0.19 ^c^
	20	61.91 ± 2.311 ^c^	4.56 ± 0.018 ^c^	1.23 ± 0.019 ^d^	9.28 ± 0.13 ^e^
Pea	10	70.71 ± 2.324 ^d^	2.76 ± 0.011 ^a^	0.71 ± 0.008 ^a^	3.32 ± 0.09 ^a^
	20	62.43 ± 0.612 ^c^	4.65 ± 0.019 ^c^	0.88 ± 0.014 ^b^	7.99 ± 0.11 ^d^

^a–d^—different letters mean significant differences between the samples within the column (*p* ≤ 0.05).
